# Digital Health–Enabled Prehospital Notification for Acute Stroke Using the Brain Saver Mobile App in Emergency Medical Transport in South Korea: Pilot Quasi-Experimental Study

**DOI:** 10.2196/92992

**Published:** 2026-10-08

**Authors:** Eunah Kim, Jeongheon Kim, Dougho Park, Suhyun Park, Byungju Lee, Mi Sun Oh, Taehee Kim, Jinhyang Kim, Gahui Gim, Joongyub Lee, Ji-Yeob Choi, Daehee Kang

**Affiliations:** 1 Institute of Health and Environment Seoul National University Seoul Republic of Korea; 2 Department of Healthcare Policy Research Korea Institute for Health and Social Affairs Sejong Republic of Korea; 3 Community Health Innovation Center Seoul National University College of Medicine Seoul Republic of Korea; 4 Department of Preventive Medicine Seoul National University College of Medicine Seoul Republic of Korea; 5 Medical Research Institute Pohang Stroke and Spine Hospital Pohang, Gyeongsangbuk-do Republic of Korea; 6 Department of Neurology Pohang St. Mary's Hospital Pohang, Gyeongsangbuk-do Republic of Korea; 7 Department of Neurology Pohang Semyeong Christianity Hospital Pohang, Gyeongsangbuk-do Republic of Korea; 8 Department of Neurology Hallym Neurological Institute Hallym University College of Medicine, Hallym University Sacred Heart Hospital Anyang, Gyeonggi-do Republic of Korea; 9 Seers Technology Co, Ltd Pyeongtaek, Gyeonggi-do Republic of Korea; 10 Integrated Major in Innovative Medical Science Seoul National University Graduate School Seoul Republic of Korea; 11 Department of Biomedical Sciences Seoul National University Graduate School Seoul Republic of Korea; 12 Institute of Health Policy and Management Seoul National University Medical Research Center Seoul Republic of Korea; 13 Medical Big Data Research Center Seoul National University Medical Research Center Seoul Republic of Korea; 14 Cancer Research Institute Seoul National University Seoul Republic of Korea; 15 BK21plus Biomedical Science Project Seoul National University College of Medicine Seoul Republic of Korea

**Keywords:** digital health, emergency medical services, mobile apps, stroke, time-to-treatment, transportation of patients

## Abstract

**Background:**

Timely access to stroke care is critical for patient prognosis. Despite the growing use of digital health strategies in stroke management, evidence supporting seamless and timely care coordination from the emergency medical services (EMS) to the emergency department (ED) remains limited. *Brain Saver*, a newly developed mobile app, addresses this gap by facilitating real-time data sharing between EMS personnel and ED clinicians during stroke transport.

**Objective:**

This study aimed to examine the potential of the Brain Saver protocol to improve existing EMS-ED workflows in emergency stroke transport by describing its functional components, implementing a pilot intervention in a real-world setting, and exploring its preliminary impact on treatment timeliness.

**Methods:**

Data were collected in Pohang, South Korea, from December 2023 to April 2024 through collaboration with 2 fire stations, eight 119 EMS and safety centers, 3 hospitals, and SEERS Technology. Preintervention training and postintervention surveys were conducted among EMS personnel (n=80) and clinicians (n=24). In this pilot, patients with stroke (*ICD-10* [*International Classification of Diseases, Tenth Revision*] codes I60-I63) in the intervention group (n=34) were transported using ambulances equipped with the Brain Saver mobile app, while the comparison group (n=129) was transported by standard ambulances to the same hospitals during the study period. With “needle” defined as any parenteral treatment, onset-to-door time and door-to-needle time were compared by group. After exact matching on age, baseline stroke severity, stroke type, and destination hospital, intervention effects were estimated using generalized estimating equations.

**Results:**

Participants (N=163) had a mean age of 69.4 (SD 15.2) years, 50.3% (82/163) were male, and 65.6% (107/163) had ischemic stroke. No significant baseline differences, including stroke severity, were observed between groups. The median onset-to-needle time was shorter in the intervention group than in the comparison group (114 vs 204 minutes; *P*=.03). In the matched analytic sample (n=91), the intervention group showed significant reductions in onset-to-needle time (Exp(β)=0.49; *P*=.009) and door-to-needle time (Exp(β)=0.67; *P*=.04) compared with the comparison group.

**Conclusions:**

Integrating digital health into emergency stroke transport may improve care timeliness by supporting real-time prehospital notification and facilitating prompt in-hospital responses. The Brain Saver mobile app shows potential use for reducing onset-to-treatment time. Further larger-scale studies are needed to evaluate implementation fidelity and scalability.

## Introduction

Rapid response at stroke onset is critical in minimizing stroke-related brain damage, reflecting the principle that “time is brain” [1]. Early transport and treatment after stroke symptom onset are associated with better outcomes, including lower in-hospital mortality [2,3] and improved functional recovery [4,5]. Accordingly, clinical guidelines for acute ischemic stroke emphasize timely care by minimizing prehospital delays and achieving treatment within 3-4.5 hours of symptom onset [6-8].

To improve access to stroke care and management, a range of digital health approaches have been developed [9]. In particular, mobile apps have demonstrated potential in reducing delays in stroke detection and clinical evaluation. For example, wearable devices paired with mobile apps can capture vital signs and detect abnormal pulse patterns, prompting timely medical attention [10]. Other applications use smartphone-based image or sensor data to identify stroke-related symptoms and activate emergency responses [11,12]. To optimize in-hospital stroke workflows, applications enabling real-time sharing of patient information among on-call stroke team members have been shown to improve interdepartmental team coordination and reduce door-to-needle time [13]. Similarly, a real-time feedback platform for paramedics and the stroke team generates automated time stamps across the stroke care continuum upon app activation, enabling more efficient monitoring of treatment timelines [14].

However, digital health strategies that explicitly integrate emergency medical services (EMS) and emergency department (ED) processes for stroke care remain limited. Although EMS-based hospital admission and direct access to specialized stroke care are key determinants of early presentation [15,16], timely transport of patients with acute stroke to appropriate facilities continues to pose a major challenge, particularly in medically underserved areas [17,18]. Notably, few studies have empirically evaluated such integrated digital interventions within real-world EMS transport settings. Therefore, further empirical evidence is needed to assess the added value of digital health technologies in improving stroke transport efficiency and rapid ED response.

To address this gap, this study implemented an intervention program using a newly developed mobile app, Brain Saver, together with a wearable electrocardiogram device (Figure 1). This app was designed with separate interfaces for paramedics and clinicians to support emergency stroke transport across EMS and ED workflows. When EMS personnel (paramedics) identify a patient with suspected stroke at the scene, they activate the app, enter patient information using 10 assessment items to generate stroke diagnostic scores, and attach a wearable electrocardiogram device. The paramedic interface provides GPS-based hospital recommendations and displays nearby hospitals with maps. After hospital selection, transport-start alerts are automatically sent to the clinician interface. During transport, clinicians can monitor patients’ symptoms, diagnostic scores, electrocardiogram data, and transport status in real time, while paramedics and clinicians communicate through messaging or video calls when needed. Detailed interfaces and functions are provided in Multimedia Appendix 1.

**Figure 1:**
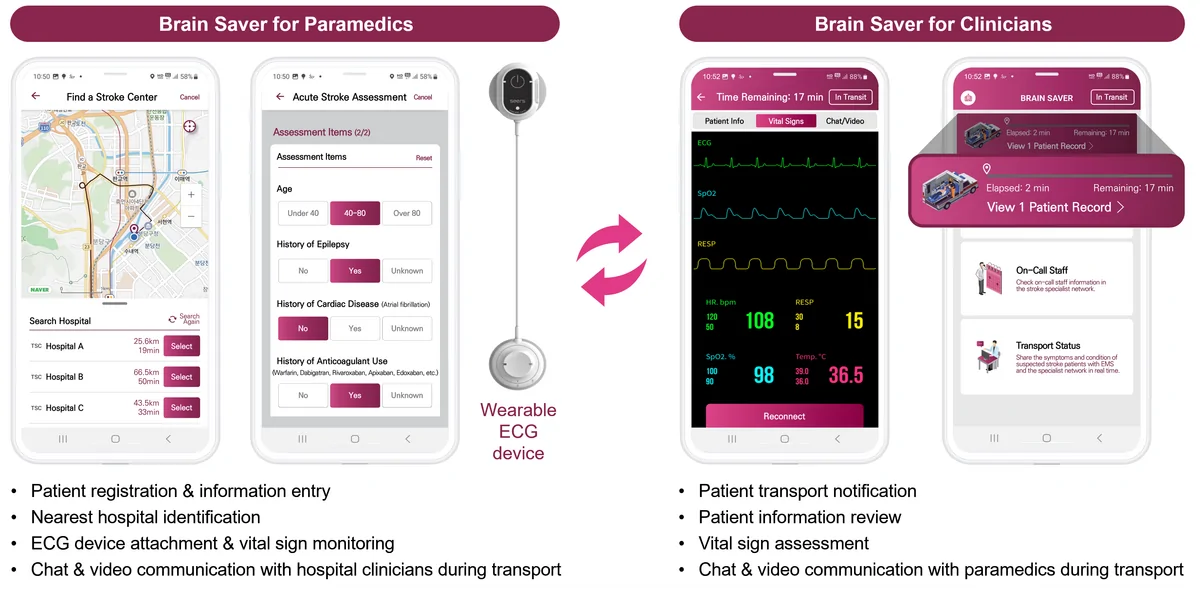
Primary functions of the Brain Saver app. When paramedics activate the app and select the nearest hospital, clinicians receive transport-start alerts and patient information through the app. ECG: electrocardiogram.

This study aimed to examine the potential of the Brain Saver protocol to improve existing EMS-ED workflows. The specific objectives were to (1) describe the development and functional components of the Brain Saver mobile app, (2) document its real-world implementation in emergency stroke transport, and (3) explore its preliminary impact on transport and treatment timeliness.

## Methods

### Digital Health Intervention: Brain Saver Protocol

This study was conducted in Pohang-si, Gyeongsangbuk-do, South Korea, through a regional Memorandum of Understanding involving Pohang Nambu and Pohang Bukbu fire stations with 2 rescue and EMS centers and six 119 safety centers under their jurisdiction, 3 hospitals, and SEERS Technology. The study comprised (1) preimplementation training, (2) a pilot phase, and (3) a postimplementation survey of app users. To ensure rigorous digital health implementation, the Guidelines and Checklist for the Reporting on Digital Health Implementations (iCHECK-DH) was applied (Multimedia Appendix 2).

### Training for EMS Personnel and Clinicians

Before the pilot demonstration, preimplementation training was provided to EMS personnel and clinicians on the use of Brain Saver to clarify roles, standardize handoffs, and reduce field-level confusion. For EMS personnel, training was delivered to 80 staff from 2 fire stations through 6 on-site sessions held from November 13-15, 2023. The curriculum covered when to initiate Brain Saver, how to register patients and enter standardized patient symptoms, how to use distance-based hospital selection, and how to attach the wearable electrocardiogram device for real-time sharing of vital signs with clinicians. For clinicians, training was provided to 24 clinicians on November 22 and 28, 2023, through 3 hospital-specific briefing sessions. Training positioned Brain Saver as a prearrival preparation support tool and focused on standardizing notification handling, patient verification, and readiness procedures. Clinicians were instructed in on-call registration and alert receipt, as well as how to review the app interface.

### Pilot Program Process

The pilot intervention program of Brain Saver–based transports was conducted from December 11, 2023, to April 5, 2024, following a standardized end-to-end process that connected EMS-side actions with clinician-side preparation (Table 1).

**Table 1:** Ten-step Brain Saver process and comparison with the conventional emergency medical services (EMS)-emergency department (ED) workflow.

Step	Activity	Responsible agent	Brain Saver workflow	Conventional EMS-ED workflow
1	Patient occurrence and EMS dispatch	Paramedics	Dispatch EMS after emergency call receipt	Dispatch EMS after emergency call receipt
2	Patient registration	Paramedics	Activate the app interface and enter on-site patient assessment information	Document on-site patient assessment records on paper-based forms
3	Hospital selection and transport	Paramedics	Select the nearest hospital based on app recommendations using GPS	Confirm hospital availability through repeated telephone-based calls until acceptance
4	Transport notification	Clinicians	Receive transport-start and 5-minute-before-arrival alerts	N/A^a^
5	Vital sign recording	Paramedics	Attach a wearable ECG^b^ device that transmits vital signs to the app in real time	Perform conventional 12-lead ECG recording and review printed ECG data
6	Prearrival information review	Clinicians	Review app-based diagnostics (Step 2) and vital signs (Step 5) before arrival	N/A
7^c^	Prearrival communication	Paramedics and clinicians	Communicate via messaging or video calls when needed	Communicate via telephone when needed
8	Preparation for patient management	Clinicians	Prepare for in-hospital management before arrival using alerts and patient information	N/A
9	Hospital arrival and handover	Paramedics	Arrive at the hospital and hand over the patient	Arrive at the hospital and hand over the patient with printed ECG data
10	Patient management	Clinicians	Initiate treatment through a preprepared in-hospital workflow	Review the printed ECG data, determine clinical care, and initiate treatment

^a^N/A: not available.

^b^ECG: electrocardiogram.

^c^Step 7 is optional and performed only when prehospital communication and treatment are needed.

For EMS personnel, the pilot workflow began at the scene when a patient was judged to be a suspected stroke case. They launched the app and recorded standardized patient information, then selected a destination hospital using distance-based recommendations, which automatically issued a transport-start alert and provided patient information to on-call clinicians. During transport, EMS personnel attached the wearable electrocardiogram device and shared vital signs through the app, and after arrival, handed over the patient to the stroke team in the ED.

For clinicians, on-call staff were provided with a dedicated Brain Saver smartphone during on-call handover. They received app-based notifications at transport initiation and again 5 minutes before arrival. Clinicians monitored incoming patients’ stroke symptoms, diagnostic scores, vital signs, and transport status in real time. When needed, they communicated with EMS personnel via messaging or video calls.

Compared with conventional EMS-ED workflows, Brain Saver provides clinicians with real-time transport notifications (Step 4) and prearrival patient information review (Step 6). These functions facilitate prearrival preparation in the ED (Step 8), whereas conventional workflows allow patient assessment only after hospital arrival without prearrival alerts. For EMS personnel, Brain Saver supports hospital selection (Step 3) and digitizes several previously paper-based or manual processes (Steps 2, 3, and 5).

A postimplementation survey of app users was conducted from April 3 to 19, 2024, to assess Brain Saver usability and to identify user needs encountered during real-world operation. The survey was distributed to all individuals who had completed the pretraining, yielding response rates of 52.5% (42/80) for EMS personnel and 54.2% (13/24) for clinicians.

### Study Design

To assess the effect of the pilot intervention program, we defined an intervention group and a comparison group based on the intervention allocation process (Figure 2). When patients with suspected stroke contacted the 119 safety reporting center, the nearest 119 safety center was dispatched according to the patient’s location, as each center operates within an assigned jurisdiction. Detailed geographic information on the study regions is provided in sections A and B in Multimedia Appendix 3.

**Figure 2:**
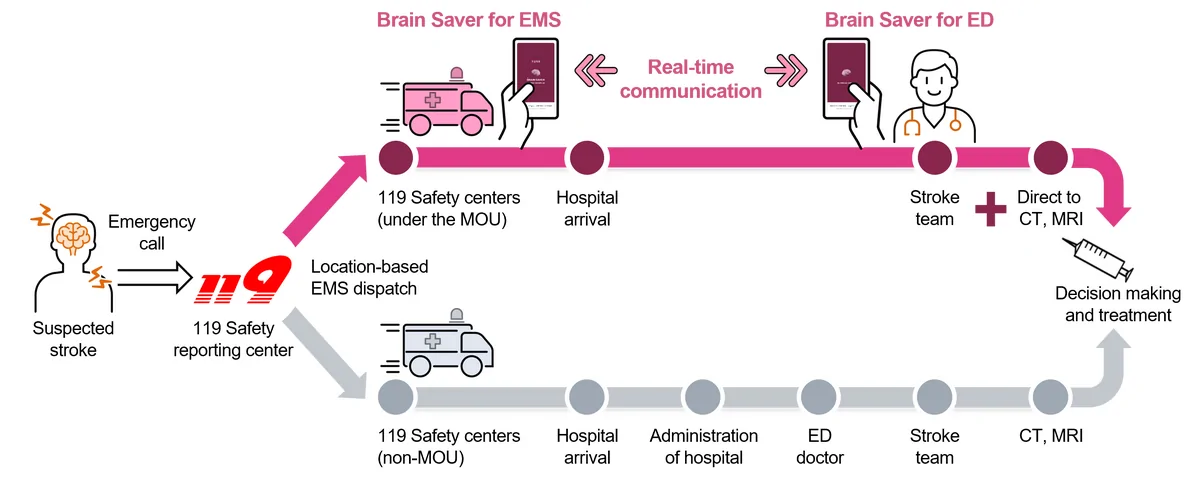
Emergency stroke transport scenarios for comparison and intervention groups. Depending on the emergency medical services (EMS) dispatch site, patients transported by Brain Saver–equipped ambulances were assigned to the intervention group, whereas those transported by conventional ambulances were assigned to the comparison group. CT: computed tomography; ED: emergency department; MOU: Memorandum of Understanding; MRI: magnetic resonance imaging.

The intervention group comprised patients with stroke transported by Brain Saver–equipped ambulances from 8 participating 119 safety centers (2 rescue and EMS and 6 safety centers) to the EDs of 3 hospitals. The comparison group consisted of patients with stroke transported to the same EDs by conventional ambulances from the remaining eight 119 safety centers or by private ambulances during the study period in Pohang-si, Korea. This group served as a nonequivalent control group without random assignment, consistent with a quasi-experimental study design.

### Study Population

We initially defined the target population as all patients with suspected stroke transported by ambulance, whereas the study population was restricted to patients with confirmed ischemic or hemorrhagic stroke (*ICD-10* codes I60-I63). A total of 425 patients with suspected stroke admitted to the 3 hospitals during the study period were identified. Patients without confirmed stroke diagnoses were present only in the comparison group (n=32) and were excluded to improve comparability between groups. Eligibility was restricted to patients transported to the ED by ambulance, excluding those admitted via outpatient clinics (n=65) or self-transport (n=124). Nonacute cases with symptom onset-to-needle times exceeding 24 hours were also excluded (n=41), consistent with the study focus on acute stroke transport. Ultimately, 163 consecutive patients comprised the analytic population (34 in the intervention and 129 in the comparison group; Figure 3).

**Figure 3:**
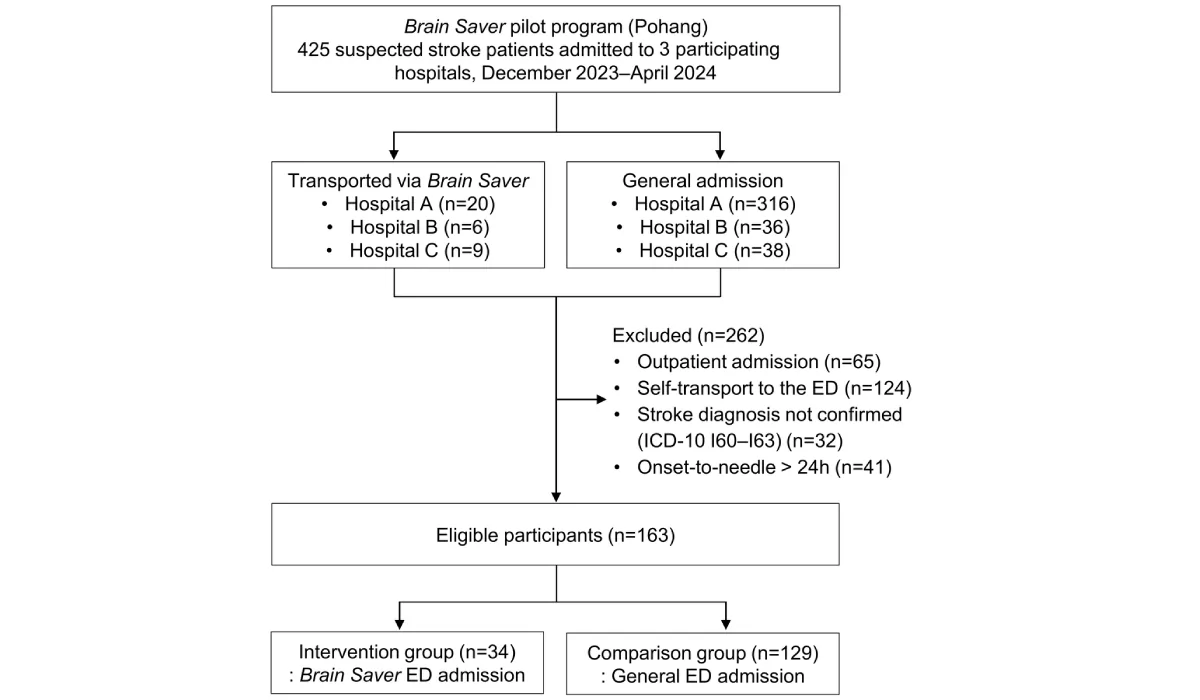
Flowchart of study population selection. Three participating hospitals in Pohang-si, South Korea, identified 425 suspected stroke admissions between December 2023 and April 2024. After exclusions, 163 ambulance-transported patients with confirmed stroke were included. ED: emergency department; ICD-10: International Classification of Diseases, Tenth Revision.

### Data Collection

The analytic dataset linked hospital electronic medical records and Brain Saver app logs. Hospital records provided sex, age, *ICD-10* diagnosis codes, National Institutes of Health Stroke Scale (NIHSS) and modified Rankin Scale (mRS) scores, treatment modalities (eg, intravenous thrombolysis—tissue plasminogen activator [tPA] and endovascular thrombectomy [EVT]), discharge outcomes, and in-hospital time stamps. App logs provided granular prehospital time intervals for the intervention group, including symptom onset, dispatch, and clinician handover times, whereas symptom onset time for the comparison group was obtained from hospital records. Despite missing data during on-site data entry into the app and during the linkage of app logs with hospital records, all available observations were retained for each analysis.

### Time Interval Measures

Time-related outcome variables were defined across two phases: (1) onset-to-door (OTD) time, defined as the interval from initial stroke symptom onset to ED arrival; and (2) door-to-needle (DTN) time, defined as the interval from ED arrival to initiation of parenteral treatment by clinicians. “Needle” in this study refers to the initiation of any parenteral treatment, including intravenous thrombolysis (eg, tPA) and other injectable treatments (eg, osmotic agents or diuretics), as the study included both patients with ischemic stroke and patients with hemorrhagic stroke. Onset-to-needle (OTN) time, encompassing both phases, represented the total time from symptom onset to treatment initiation. Length of hospital stay (LOS) was also evaluated as a measure of care quality and operational efficiency in early stroke management [19].

During data cleaning, logically implausible values (eg, 0 minutes for OTD time) and extreme outliers identified using the 3×IQR rule after log transformation of time variables were treated as missing values, as detailed in section C in Multimedia Appendix 3.

### Statistical Analyses

To evaluate the effect of the pilot intervention program, characteristics between the intervention and comparison groups were compared using Fisher exact test or Pearson chi-square test for categorical variables, Welch *t* test for mean differences, and the Wilcoxon rank-sum test for median differences. To compare the cumulative incidence of discharge between groups over time, adjusted Fine-Gray competing risks regression analyses were performed to estimate subdistribution hazard ratios (SHRs). In-hospital death was treated as a competing event and transfer to tertiary hospitals as a censoring event. Sex, age, NIHSS score at admission, stroke type, and destination hospital were included as covariates.

To enhance comparability between groups given the limited sample size, we used an exact matching approach on key covariates. Matching strata were defined by age (<65 and ≥65 years), baseline NIHSS category at admission (0, 1-4, 5-15, and 16-42), stroke subtype (ischemic and hemorrhagic), and destination hospital (A, B, or C). All eligible comparison patients within each stratum were retained, yielding a many-to-many matched design. Due to missing baseline NIHSS data and unmatched strata, the analytic sample was restricted to 91 patients (19 intervention and 72 comparison). With individuals contributing repeatedly within strata, the analysis included 271-309 paired observations across models. To account for within-stratum correlation induced by this repeated contribution, we fitted generalized estimating equations (GEE) with a robust variance estimator, modeling log-transformed outcomes with a Gaussian distribution and an identity link. All analyses were performed using SAS (version 9.4; SAS Institute Inc) and R software (version 4.4.2; R Foundation for Statistical Computing).

### Ethical Considerations

The research protocol was approved by the institutional review boards of the 3 hospitals that participated in the pilot program: Pohang Stroke and Spine Hospital (IRB number PSSH0475-202405-HR-009-01); Pohang St Mary’s Hospital (IRB number 0749-240603-HR-075-01); and Pohang Semyeong Christianity Hospital (IRB number PSMCHIRB-2024-25). Because patient data were collected and analyzed in an anonymized form, the requirement for informed consent was waived.

Regarding patient information security, the Brain Saver app was designed to protect patient privacy throughout data collection, storage, and transmission processes. All collected data were deidentified before storage and transmission to minimize the risk of patient identification. Transmitted data were protected using encrypted communication protocols (eg, Transport Layer Security), reducing the risk of external interception or unauthorized access. The system complied with the Personal Information Protection Act and the Medical Service Act of South Korea.

## Results

The geographic and temporal distribution of intervention cases during the pilot program are presented in sections A and B in Multimedia Appendix 3. The postimplementation survey of app users indicated that EMS personnel and clinicians valued key operational functions of Brain Saver, particularly access to patient assessment results, distance-based hospital recommendations, and transport status tracking (Table 2). In contrast, 36% (15/42) of EMS personnel reported difficulty entering information into the app and suggested additional functions for electrocardiogram storage and clinician confirmation of transport alerts. Clinicians suggested enhancing the basic patient information displayed in the app and reducing frequent alerts.

**Table 2:** Findings from the Brain Saver user experience survey among emergency medical services (EMS) personnel and clinicians^a^.

Survey item		Key findings
EMS personnel (n=42)		
	Most satisfying functions (top 3)^b^	Diagnostic result display (30%) Distance-based hospital recommendation (18%) Automatic current-location entry (17%)
	User experience with app functions	Chat and video: 7 (17%) respondents used the messaging function during transport to share biological information other than ECGc data, provide estimated arrival times, and confirm EDd availability. Posttransport feedback: 35 (83%) respondents reported positive perceptions of the final diagnosis confirmation function after transport
	Difficulties in data input	15 (36%) respondents reported difficulty entering the patient information
	Additional improvement requests	A function for storing ECG data in the app A feedback mechanism indicating whether clinicians have checked transport-related information Integration with existing fire-service systems to reduce duplicated work
Clinicians (n=13)		
	Most satisfying functions (top 3)^b^	Transport status tracking via the status bar (40%) Viewing the patient diagnostic score (31%) Viewing the on-call clinician list within the same hospital (10%)
	User experience with app functions	Real-time ECG sharing: 11 (85%) received notifications; 10 reported it supported clinical decision-making Arrhythmia alarm: 5 (38%) received alerts; 4 reported it supported clinical decision-making
	Additional improvement requests	Improving basic patient information Addressing workflow burden caused by frequent arrhythmia alerts Clarifying legal issues related to patient privacy and medical liability

^a^The survey was distributed to all personnel who completed the pretraining; response rates were 52.5% (42/80) for EMS personnel and 54.2% (13/24) for clinicians.

^b^Percentages are each feature’s share of the total weighted score, with first, second, and third place weighted 100, 70, and 30, respectively. See Multimedia Appendix 4 for details.

^c^ECG: electrocardiogram.

^d^ED: emergency department.

Table 3 presents the descriptive characteristics of the intervention (n=34) and comparison (n=129) groups. No statistically significant differences were observed in overall characteristics between the 2 groups. All participants (N=163) were 50.3% (n=82) men and had a mean age of 69.4 (SD 15.2) years; 65.6% (n=107) were diagnosed with ischemic stroke and 34.4% (n=56) with hemorrhagic stroke; 17% (16/94) of patients with ischemic stroke received intravenous tPA, and 28.9% (26/90) underwent EVT. At admission, NIHSS-based stroke severity did not differ significantly between groups (median 9, IQR 5-12 vs median 6, IQR 2-12; *P*=.12). Discharge NIHSS scores and mRS (a measure of functional outcome) also showed no significant differences. Regarding the discharge destination, 27.3% (6/22) of patients in the intervention group were discharged directly to home, compared with 45% (58/129) in the comparison group (*P*=.12).

**Table 3:** Descriptive results of the intervention and comparison groups. Denominators below the total study population (N=163) reflect missing data identified during the linkage of hospital records.

			Total (N=163)		Intervention (n=34)		Comparison (n=129)		*P* value^a^	
Sex (N=163), n (%)										.26
	Male	82 (50.3)		20 (58.8)		62 (48.1)				
	Female	81 (49.7)		14 (41.2)		67 (51.9)				
Age in years (N=163), mean (SD)			69.4 (15.2)		69.3 (15.8)		69.4 (15.1)		.97	
Hospital ID (N=163), n (%)										<.001
	A	138 (84.7)		19 (55.9)		119 (92.2)				
	B	13 (8.0)		6 (17.6)		7 (5.4)				
	C	12 (7.4)		9 (26.5)		3 (2.3)				
Stroke type (N=163), n (%)										.50
	Hemorrhagic stroke (I61-I62)	56 (34.4)		10 (29.4)		46 (35.7)				
	Ischemic stroke (I63)	107 (65.6)		24 (70.6)		83 (64.3)				
Treated: tPA^b,c^ (n=94), n (%)										.73
	Yes	16 (17.0)		2 (11.8)		14 (18.2)				
	No	78 (83.0)		15 (88.2)		63 (81.8)				
Treated: EVT^b,d^ (n=90), n (%)										>.99
	Yes	26 (28.9)		2 (25.0)		24 (29.3)				
	No	64 (71.1)		6 (75.0)		58 (70.7)				
NIHSS^e^ at admission (0-42^f^; n=142), median (minimum-maximum)			7 (0-33)		9 (1-30)		6 (0-33)		.12	
NIHSS at discharge (0-42^f^; n=131), median (minimum-maximum)			3 (0-34)		6 (0-34)		2 (0-34)		.11	
mRS^g^ at discharge (0-6; n=147), n (%)										.55
	Favorable outcome (0-2)	66 (44.9)		9 (39.1)		57 (46.0)				
	Unfavorable outcome (3-6)	81 (55.1)		14 (60.9)		67 (54.0)				
Discharge destination (n=151), n (%)										.12
	Home	64 (42.4)		6 (27.3)		58 (45.0)				
	Rehabilitation facility/nursing home/long-term care hospital	62 (41.1)		9 (40.9)		53 (41.1)				
	Tertiary hospital	14 (9.3)		4 (18.2)		10 (7.8)				
	In-hospital death	11 (7.3)		3 (13.6)		8 (6.2)				

^a^Categorical variables were compared using the Fisher exact test or Pearson chi-square test, depending on sample size. Median differences were compared using the Wilcoxon rank-sum test, and mean differences using the Welch *t* test.

^b^Percentages for tissue plasminogen activator and endovascular thrombectomy were calculated only among patients with ischemic stroke and available treatment data, after excluding cases with missing treatment data.

^c^tPA: tissue plasminogen activator.

^d^EVT: endovascular thrombectomy.

^e^NIHSS: National Institutes of Health Stroke Scale.

^f^NIHSS scores at hospital admission and discharge were presented only for patients who survived, ensuring that the same study population was compared at both time points.

^g^mRS: modified Rankin Scale.

Table 4 summarizes the timeline from symptom onset to discharge. Overall, the median OTD, DTN, and OTN times were 88 (IQR 48-273), 58 (IQR 39-98), and 177 (IQR 100-426) minutes, respectively, and the median LOS was 19 (IQR 10-29) days. The intervention group had a significantly shorter median OTN time than the comparison group (114, IQR 82-258 minutes vs 204, IQR 105-460 minutes; *P=*.03).

**Table 4:** Timeline from symptom onset to hospital discharge among patients with stroke in intervention and comparison groups.

			Total (N=163)		Intervention (n=34)		Comparison (n=129)		*P* value^a^
Onset-to-door time (A), n			155		29		126		
	Minutes, mean (SD)	230.9 (300.8)		253.6 (350.2)		225.6 (289.6)		.69	
	Minutes, median (minimum-maximum)	88 (19-1438)		58 (19-1102)		105 (20-1438)		.18	
Door-to-needle time (B)^b^, n			153		28		125		
	Minutes, mean (SD)	125.7 (220.1)		74.7 (57.0)		137.1 (240.7)		.01	
	Minutes, median (minimum-maximum)	58 (3-1248)		52 (8-234)		59 (3-1248)		.74	
Onset-to-needle time (A+B)^b^, n			152		26		126		
	Minutes, mean (SD)	331.1 (340.6)		254.3 (306.0)		347.0 (346.3)		.18	
	Minutes, median (minimum-maximum)	177 (25-1390)		114 (26-999)		204 (25-1390)		.03	
Length of stay among patients discharged alive^c^, n			131		18		113		
	Day, mean (SD)	23.0 (16.9)		19.9 (15.4)		23.5 (17.2)		.38	
	Day, median (minimum-maximum)	19 (1-89)		16 (1-58)		20 (2-89)		.39	

^a^Median differences were compared using the Wilcoxon rank-sum test, and mean differences using the Welch *t* test.

^b^“Needle” refers to overall injectable treatment provision across both stroke subtypes.

^c^Length of stay was estimated among patients discharged alive after excluding 11 in-hospital deaths, 14 transfers to tertiary hospitals, and cases with missing data.

More detailed time-interval data were available only for the intervention group (section D in Multimedia Appendix 3), with median times of 7 (IQR 4-9) minutes from dispatch to hospital arrival and 6 (IQR 4-8) minutes from arrival to clinician handover. By stroke subtype (section E in Multimedia Appendix 3), patients with ischemic stroke had a median OTN time of 223 (IQR 118-460) minutes, and 59.6% (59/99) achieved an OTN time within the clinically relevant 4.5-hour treatment window.

Table 5 presents the cumulative incidence of discharge over time by group, accounting for competing risks and censoring. A higher NIHSS score at admission was associated with delayed discharge (SHR=0.94 per 1-point increase; *P*<.001), whereas the intervention was not significantly associated with the timing of discharge (SHR=0.78; *P*=.37).

**Table 5:** Fine-Gray competing risks regression of discharge cumulative incidence^a^.

	β	Exp(β), SHR^b^ (95% CI)	SE	Z value	*P* value
Intervention (reference: comparison)	–.25	0.78 (0.46-1.34)	0.28	–0.89	.37
Age	–.00	1.00 (0.99-1.01)	0.01	–0.47	.64
Female (reference: male)	–.09	0.91 (0.62-1.35)	0.20	–0.47	.64
NIHSS^c^ score at admission	–.06	0.94 (0.92-0.97)	0.01	–4.60	<.001
Ischemic stroke (reference: hemorrhagic stroke)	.28	1.33 (0.91-1.95)	0.19	1.47	.14
Hospital B (reference: Hospital A)	–.79	0.45 (0.12-1.74)	0.69	–1.16	.25
Hospital C (reference: Hospital A)	–.32	0.72 (0.33-1.57)	0.40	–0.82	.41

^a^In-hospital death was treated as a competing event and transfer to tertiary hospitals as a censoring event.

^b^SHR: subdistribution hazard ratio.

^c^NIHSS: National Institutes of Health Stroke Scale.

Table 6 presents the intervention effects on transport time and length of stay in a sample matched on age, baseline NIHSS score category, stroke type, and destination hospital. The intervention group showed a statistically significant 33% reduction in DTN time (Exp(β)=0.67; *P*=.04) and a 51% reduction in OTN time (Exp(β)=0.49; *P*=.009). The intervention group also showed a 22% reduction in LOS compared with the comparison group (Exp(β)=0.78; *P*=.007) in this matched sample, which adjusted for potential confounders.

**Table 6:** Effects of Brain Saver intervention on transport time and length of stay: generalized estimating equation models (GEE) analyses using a many-to-many matched sample.a

GEE with a normal distribution and identity link	Intervention vs comparison (reference)			
	Estimate, β	Exp(β)^b^	Z value	*P* value
Model 1: Y=ln(Onset-to-door time)	–0.62	0.54	–1.88	.06
Model 2: Y=ln(Door-to-needle time)	–0.40	0.67	–2.02	.04
Model 3: Y=ln(Onset-to-needle time)	–0.72	0.49	–2.61	.009
Model 4: Y=ln(Length of stay among patients discharged alive)	–0.25	0.78	–2.72	.007

^a^Each GEE model accounted for matching strata defined by age category, National Institutes of Health Stroke Scale (NIHSS) category at admission, stroke subtype, and destination hospital. Due to missing baseline NIHSS data and unmatched strata, the analytic sample was restricted to 91 patients (19 intervention and 72 comparison). Through many-to-many matching across 10 matching strata, these 91 patients contributed 293 (model 1), 309 (model 2), 290 (model 3), and 271 (model 4) observations to the GEE analyses, respectively.

^b^Exponentiated estimates are reported for log-transformed continuous outcomes.

## Discussion

### Principal Findings

The Brain Saver mobile app was integrated into standard EMS and ED workflows and streamlined operational processes, with generally positive user feedback. Through real-time collaboration between paramedics and clinicians, the app supports nearest-hospital navigation and emergency alert activation, enabling rapid in-hospital response upon patient arrival based on app-entered data. This prehospital intervention facilitates timely access to stroke care, potentially reducing OTN time.

EMS personnel and clinicians identified the app-based prenotification process as the most useful component for situational awareness and decision-support. Given that documentation burden in clinical settings can hinder the development of shared situational awareness [20], the app-based intuitive presentation and transfer of information may support clear and concise communication, thereby enhancing continuity of care. Moreover, prior evidence indicates that cognitive aids, such as checklists and decision-support tools, can reduce missed care and improve accuracy, particularly in clinical emergencies [21]. In this context, Brain Saver functions, such as tracking transport status and summarizing stroke assessment scores, may serve as meaningful clinical decision-support tools. In addition, digitalized recording of electrocardiogram data and patient information through the app may improve EMS-ED workflow efficiency by replacing paper-based and manual processes.

The principal finding of this pilot study was that the intervention group experienced shorter OTN times in both unadjusted median comparisons and matched-sample analyses. This improvement may be driven more by reduced DTN time than by OTD time, because DTN time showed significant GEE-estimated reductions, whereas OTD time did not differ significantly in any analysis. The core function of Brain Saver, which provides prenotification and patient information during transport, may facilitate hospital preparedness before patient arrival. Previous studies have similarly shown that, among the various advantages of EMS transport, prenotification systems in particular facilitate more rapid in-hospital evaluation by reducing door-to-imaging time, which in turn leads to shorter DTN and OTN times [22-26].

No significant difference in OTD time was observed between groups. A previous study using a real-time feedback mobile app for stroke emergency management similarly reported no significant effect on OTD time following the intervention [27]. One plausible explanation relates to the study design, in which both groups consisted of patients transported by EMS, which itself is a strong predictor of early hospital arrival [15,28]. Moreover, the findings of this study suggest that OTD time may have been largely determined by symptom onset-to-dispatch time, given that dispatch-to-hospital arrival times in the intervention group were relatively short, ranging from only 3-18 minutes (section D in Multimedia Appendix 3).

Regarding LOS, the findings warrant cautious interpretation. Although mean and median LOS and cumulative discharge patterns did not differ significantly between groups, GEE analyses in the matched sample estimated a shorter overall LOS in the intervention group. This discrepancy may reflect differences in discharge timing distributions and baseline stroke severity between groups. The comparison group included relatively more patients with mild stroke and showed faster early discharge but more extreme LOS values, whereas the intervention group showed generally slower discharge with fewer extreme values (section F in Multimedia Appendix 3). Previous studies have suggested that shorter LOS may reflect higher quality of care during the early phase of stroke management [19], while the association between stroke severity and LOS may be nonlinear or bidirectional depending on severity level [29]. Definitive conclusions cannot be drawn from this small sample, and larger studies are needed to improve generalizability.

### Study Strengths

Drawing on real-world deployment, this study provides empirical evidence supporting the expanding role of digital health in seamless care transitions for stroke transport. We adopted a quasi-experimental study design and incorporated a structured familiarization phase to support adherence to standardized end-to-end protocols. This approach enhances data reliability and partially strengthens causal inference compared with previous cross-sectional [12] or retrospective observational studies [27,30] that addressed similar research objectives. In addition, we captured the complete care timeline from symptom onset to hospital discharge, extending beyond prior studies focused primarily on postadmission phases [13,14,30].

Notably, the Brain Saver intervention also has important practical implications. This pilot program aligns with best-practice strategies for stroke quality improvement, including “EMS prenotification, rapid triage and stroke team notification protocols, single-call activation, and a team-based approach” [25,31,32], and extends their implementation through digital integration. Our findings suggest the added value of strengthening continuity across the EMS-ED interface, beyond established benefits of mobile and wearable technologies in stroke care [33,34]. Such coordinated processes may support shared decision-making and facilitate timely stroke care.

### Limitations

There are several limitations. First, the definitions of the study population and time-to-needle variables should be interpreted with caution. We included both patients with ischemic stroke and patients with hemorrhagic stroke and defined “needle” time as the initiation of any parenteral treatment, whereas most previous studies have focused exclusively on patients with ischemic stroke receiving tPA-specific therapy. Second, several primary variables had substantial missing data. Some symptom-onset data were missing in the intervention group during on-site data entry into the app. Missing in-hospital treatment data were likely due to the fragmented data-linkage process between app logs and hospital records. In addition, logical errors in the OTD time (eg, 0 minutes) were identified in the comparison group, whose data were derived from hospital records. These data quality issues emerged during the 4-month real-world pilot implementation involving multiple agencies and hospitals. Third, detailed prehospital time intervals were available only for the intervention group, limiting comparability between groups. Fourth, the absence of nonconfirmed stroke cases in the intervention group may reflect a field-level selection mechanism, whereby the app was initiated preferentially for clinically apparent stroke presentations. Fifth, given the quasi-experimental study design with a newly adopted device, the Hawthorne effect—heightened performance under observation—cannot be entirely excluded and may have contributed to the shorter treatment times observed in the intervention group. Finally, the matched analytic sample was limited (n=91 in the GEE model, and fewer in the LOS analysis), constraining statistical power and the generalizability of the results.

### Future Work

This pilot study provides preliminary evidence on the feasibility of applying a newly developed digital health intervention in real-world EMS-ED workflows. Given the small sample, larger studies are needed to improve comparability and generalizability of the findings. Future studies could investigate broader populations and more detailed prehospital time intervals, including patients with suspected stroke or those with OTN times exceeding 24 hours, which were beyond the scope of this study.

Regarding app functionality, app users in this pilot study suggested improvements to the alert system, particularly reducing alert frequency for clinicians and providing EMS personnel with feedback on whether clinicians had acknowledged the alerts. In addition, the criteria for app activation (ie, those determining inclusion in the intervention group) warrant further refinement to cover less typical or ambiguous stroke presentations.

To optimize EMS-ED workflows, interoperability challenges within existing EMS systems remain an important concern [35]. As our approach relies on an app-based digital system, transitioning ambulance documentation from paper to digital systems will be crucial. The app could initially serve as a complement to existing workflows and, through stepwise implementation, eventually function as a complete substitute for paper-based workflows. Reflecting the app-user survey, efforts to minimize duplicated documentation tasks during this transition will be important.

### Conclusions

This formative, quasi-experimental pilot study suggests that Brain Saver can be integrated into routine EMS and ED workflows for stroke transport. By facilitating real-time prenotification and prearrival preparation, Brain Saver was associated with shorter OTN time. App-user feedback further supported the feasibility of the intervention among EMS personnel and clinicians, emphasizing the value of rapid triage and shared situational awareness. Taken together, these findings highlight the potential of digital health interventions to enhance coordinated EMS-ED workflows and improve timely stroke treatment across the care continuum.

## Data Availability

The datasets generated during this study are not publicly available due to the potential risk of reidentification of participating hospitals, but are available from the corresponding author upon reasonable request.

## Appendix

Multimedia Appendix 1 Brain Saver app interfaces.

Multimedia Appendix 2 iCHECK-DH checklist.

Multimedia Appendix 3 Pilot implementation process and outcomes.

Multimedia Appendix 4 Brain Saver user survey results.

## Abbreviations

DTN: door-to-needle
ED: emergency department
EMS: emergency medical services
EVT: endovascular thrombectomy
GEE: generalized estimating equations
ICD-10: International Classification of Diseases, Tenth Revision
iCHECK-DH: Guidelines and Checklist for the Reporting on Digital Health Implementations
LOS: length of hospital stay
mRS: modified Rankin Scale
NIHSS: National Institutes of Health Stroke Scale
OTD: onset-to-door
OTN: onset-to-needle
SHR: subdistribution hazard ratio
tPA: tissue plasminogen activator
