# Supplementary material for: Digital Health–Enabled Prehospital Notification for Acute Stroke Using the Brain Saver Mobile App in Emergency Medical Transport in South Korea: Pilot Quasi-Experimental Study
Source: JMIR Med Inform. 2026 Oct 8;14:e92992. doi: 10.2196/92992 (PMC13691466; doi:10.2196/92992)
Supplement: Supplementary file 1 [file medinform_v14i1e92992_app1.docx]

This is a Multimedia Appendix to a full manuscript published in the *JMIR Medical Informatics* For full copyright and citation information see <http://dx.doi.org/10.2196/xxxxx>

**Brain Saver Application Interfaces**

The Brain Saver application is designed to support decision-making for hospital selection and real-time patient information sharing between EMS personnel and clinicians during the transport of patients with suspected stroke. The application consists of separate interfaces for paramedics and clinicians, whose main features are presented in Figures S1 and S2, respectively.

When paramedics activate the app, the GPS system automatically captures the dispatch location and identifies the nearest hospital. EMS personnel attach a wearable device to patients for vital sign monitoring and assess patients’ clinical status by selecting yes/no responses to 10 assessment items displayed on the app interface (Table S1 and Figure S1). The app then automatically calculates the corresponding STROKE and ELVO scores in real time.

Clinicians at the destination hospital receive a notification when transport is initiated and are provided with patient information in advance through the app, including the patient’s sex, age category, responses to the 10 assessment items, calculated STROKE and ELVO scores, and vital signs. Clinicians receive an additional notification five minutes before the estimated arrival time calculated by the GPS system.

| 1. App activation, patient registration, and diagnosis | | 2. Nearest hospital selection and transport initiation | | 3. Patient information review and verification |
| --- | --- | --- | --- | --- |
|  | |  | |  |
|  |  | |  | |
| 4. ECG attachment and vital sign monitoring | 5. Real-time chat and video communication | | 6. Transport completion and mission status update | |
|  |  | |  | |

**Figure S1.** Main features of the Brain Saver application interface for paramedics

| 1. Transport initiation notification | | 2. Transport status verification | | 3. Patient information review and verification |
| --- | --- | --- | --- | --- |
|  | |  | |  |
|  |  | |  | |
| 4. Vital sign monitoring | 5. Real-time chat and video communication | | 6. Five-minute pre-arrival notification | |
|  |  | |  | |

**Figure S2.** Main features of the Brain Saver application interface for clinicians

**Table S1.** Diagnostic assessment items at Brain Saver app interface for EMS personnel

| **No.** | **Assessment item** | **Response options** |
| --- | --- | --- |
| 1 | History of epilepsy | No / Yes / Unknown |
| 2 | History of cardiac disease (atrial fibrillation) | No / Yes / Unknown |
| 3 | History of anticoagulant use | No / Yes / Unknown |
| 4 | Abnormal blood glucose level (<80 or >400 mg/dL) | No / Yes / Unknown |
| 5 | Decreased level of consciousness | No / Yes / Unknown |
| 6 | Gaze deviation | No / Yes |
| 7 | Facial palsy | No / Yes |
| 8 | Arm or leg weakness/paralysis | No / Yes |
| 9 | Dysarthria or language impairment | No / Yes |
| 10 | Age group | <40 / 40–80 / >80 years |
