# Supplementary material for: Digital Health–Enabled Prehospital Notification for Acute Stroke Using the Brain Saver Mobile App in Emergency Medical Transport in South Korea: Pilot Quasi-Experimental Study
Source: JMIR Med Inform. 2026 Oct 8;14:e92992. doi: 10.2196/92992 (PMC13691466; doi:10.2196/92992)
Supplement: Supplementary file 3 [file medinform_v14i1e92992_app3.docx]

This is a Multimedia Appendix to a full manuscript published in the *JMIR Medical Informatics* For full copyright and citation information see <http://dx.doi.org/10.2196/xxxxx>

**Pilot Implementation Process and Outcomes**

**Appendix A.** Geographic information on the pilot program implementation	**2**

**Appendix B.** Daily enrollment of the study population	**4**

**Appendix C.** Data cleaning procedures for time-related variables	**6**

**Appendix D.** Detailed prehospital and in-hospital timeline of the intervention group	**7**

**Appendix E.** Timeline from transport to discharge by stroke subtype	**8**

**Appendix F**. Distribution of length of stay by group	**11**

**Appendix A. Geographic information on the pilot program implementation**

Figure S1 presents the locations of fire and safety centers and participating hospitals in Pohang-si, South Korea. Two EMS centers and six 119 Safety Centers were designated as participating agencies and transported suspected stroke patients using Brain Saver app–equipped ambulances (intervention group). The remaining eight 119 Safety Centers served as non-participating centers and transported patients using the conventional ambulance system (comparison group).

Participating centers were selected based on EMS service demand and the feasibility of stable pilot implementation. Although each 119 Safety Center is assigned to a specific administrative jurisdiction, EMS centers operate as regional command units supervising multiple local centers and serving broader northern or southern areas of Pohang-si. This operational structure likely minimized regional selection bias between the intervention and comparison groups.

**Figure S1**. Locations and administrative jurisdictions of participating 119 EMS and Safety Centers and the three participating hospitals in Pohang-si, South Korea

Figure S2 illustrates the geographical distribution of ambulance dispatch locations and their corresponding hospital destinations for the intervention group. Each red dot represents one of the 34 intervention cases.

**Source**: Authors’ own elaboration.

**Note:** Spatial analyses to visualize the pilot program implementation were performed using QGIS (version 3.4). The road network data were retrieved from OpenStreetMap (OSM) and overlaid using the XYZ tile method in QGIS software based on EPSG:4326 coordinates. Yellow regions indicate Pohang administrative boundaries; red dots represent ambulance dispatch sites; crosses denote the three hospitals. Only 30 of the 34 pilot cases are shown due to missing information.

**Figure S2.** Ambulance dispatch sites and corresponding hospital destinations in the intervention group

**Appendix B. Daily enrollment of the study population**

Figure S1 presents the number of stroke patients admitted via the emergency department at three hospitals during the study period, which was conducted in the winter season in Korea. Intervention cases were most frequent in January and March 2024, while comparison cases peaked in December 2023 (Figure S3). However, daily cumulative percentages were generally similar between groups (Figure S4). Because both groups were enrolled during the same period within the same hospitals, the balanced registration pattern may have minimized potential seasonal effects on group comparisons.

**Figure S3** Daily distribution of enrolled patients by group

**Figure S4.** Daily cumulative percentage of enrolled patients by group

**Appendix C. Data cleaning procedures for time-related variables**

We cleaned the time-related variables. First, non-emergency patients with time values exceeding 1,440 minutes during any transport or in-hospital phase were excluded, as they fell outside the target study population. Second, logically implausible values and extreme outliers identified using the 3×IQR rule were treated as missing rather than excluding the entire observation. Specifically, cases with 0 minutes from symptom onset to hospital arrival (n=2; both in the comparison group) were treated as missing. After log transformation of each time variable, hospital arrival-to-treatment times of ≤2 minutes (n=3; one in the comparison and two in the intervention group) were identified as extreme outliers and treated as missing. The final distributions of the time intervals are presented in Figure S5.

**Figure S5.** Distributions of raw (upper) and log-transformed (lower) time interval variables after data cleaning

**Appendix D. Detailed prehospital and in-hospital timeline of the intervention group**

**Table S1.** Distribution of time intervals during EMS-ED transport in the intervention group (N = 34)

| (Unit: minutes; LOS in days) | **N** | **Mean (SD)** | | **Distribution** | | | | |
| --- | --- | --- | --- | --- | --- | --- | --- | --- |
|  |  |  |  | **Min** | **Q1** | **Median** | **Q3** | **Max** |
| **Onset-to-door time (A)** | 29 | 253.6 | (350.2) | 19 | 33 | 58 | 356 | 1102 |
| Onset-to-dispatch | 27 | 264.4 | (357.7) | 13 | 34 | 53 | 380 | 1097 |
| Dispatch-to-door | 30 | 7.3 | (3.6) | 3 | 4 | 7 | 9 | 18 |
| **Door-to-needle time (B)** | 28 | 74.7 | (57.0) | 8 | 45 | 52 | 95 | 234 |
| Door-to-clinician | 32 | 6.8 | (5.1) | 0 | 4 | 6 | 8 | 24 |
| Clinician-to-needle | 27 | 70.7 | (56.2) | 9 | 41 | 49 | 82 | 230 |
| **Onset-to-needle time (A+B)** | 26 | 254.3 | (306.0) | 26 | 82 | 114 | 258 | 999 |
| **Length of stay ^a^** | 18 | 19.9 | (15.4) | 1 | 7 | 16 | 30 | 58 |

**^a^** Length of stay was estimated among patients discharged alive after excluding in-hospital deaths, transfers to tertiary hospitals, and cases with missing data.

**Appendix E. Timeline from transport to discharge by stroke subtype**

**Table S2.** Time intervals among patients with ischemic stroke

|  | **Total** | **Intervention** | **Comparison** | ***P* value** **^a^** |
| --- | --- | --- | --- | --- |
|  | **(N = 107)** | **(N = 24)** | **(N = 83)** |  |
| **Onset-to-door time (min)** |  |  |  |  |
| n | 101 | 20 | 81 |  |
| Mean (SD) | 232.8 (282.1) | 224.8 (326.7) | 234.8 (272.3) | .90 |
| Median [Min, Max] | 108 [19, 1102] | 58 [19, 1102] | 118 [21, 1081] | .17 |
| **Door-to-needle time (min)** |  |  |  |  |
| n | 102 | 20 | 82 |  |
| Mean (SD) | 151.7 (254.5) | 75.6 (54.9) | 170.3 (279.7) | .005 |
| Median [Min, Max] | 68 [8, 1248] | 52 [8, 234] | 69 [27, 1248] | .14 |
| **Onset-to-needle time (min)** |  |  |  |  |
| n | 99 | 17 | 82 |  |
| Mean (SD) | 365.2 (352.4) | 186.5 (203.1) | 402.3 (366.1) | .002 |
| Median [Min, Max] | 223 [28, 1390] | 105 [50, 880] | 245 [28, 1390] | .002 |
| **Length of stay (day)^b^** |  |  |  |  |
| n | 89 | 14 | 75 |  |
| Mean (SD) | 20.4 (14.0) | 17.9 (12.9) | 20.8 (14.3) | .45 |
| Median [Min, Max] | 19 [1, 71] | 16 [1, 39] | 19 [2, 71] | .49 |

**^a^** Wilcoxon rank-sum test and Welch’s two sample t-test were used for comparisons of medians and means, respectively.

**^b^** Length of stay was estimated among patients discharged alive after excluding in-hospital deaths, transfers to tertiary hospitals, and cases with missing data.

**Table S3.** Proportion of patients with ischemic stroke arriving within the eligible time window

|  | | **N** | **Total** | | **Intervention** | | **Comparison** | | ***P* value ^a^** |
| --- | --- | --- | --- | --- | --- | --- | --- | --- | --- |
|  |  |  | **(N = 107)** | | **(N = 24)** | | **(N = 83)** | |  |
|  |  |  | **n** | **(%)** | **n** | **(%)** | **n** | **(%)** |  |
| **Onset-to-door time** | | 101 |  |  |  |  |  |  | .43 |
|  | Time ≤ 3 hours (180 min) |  | 63 | (62.4) | 14 | (70.0) | 49 | (60.5) |  |
|  | Time > 3 hours (180 min) |  | 38 | (37.6) | 6 | (30.0) | 32 | (39.5) |  |
| **Door-to-needle time** | | 102 |  |  |  |  |  |  | .09 |
|  | Time ≤ 60 minutes |  | 44 | (43.1) | 12 | (60.0) | 32 | (39.0) |  |
|  | Time > 60 minutes |  | 58 | (56.9) | 8 | (40.0) | 50 | (61.0) |  |
| **Onset-to-needle time** | | 99 |  |  |  |  |  |  | .04 |
|  | Time ≤ 4.5 hours (270 min) |  | 59 | (59.6) | 14 | (82.4) | 45 | (54.9) |  |
|  | Time > 4.5 hours (270 min) |  | 40 | (40.4) | 3 | (17.6) | 37 | (45.1) |  |

**^a^** Pearson’s chi-squared test was used to compare categorical variables between groups.

Note: Clinical guidelines do not specify an eligibility window for onset-to-door time, whereas recommended targets include a door-to-needle time within 60 minutes and an onset-to-needle time within 3 to 4.5 hours of symptom onset.

**Table S4.** Time intervals among patients with hemorrhagic stroke

|  | **Total** | **Intervention** | **Comparison** | ***P* value^a^** |
| --- | --- | --- | --- | --- |
|  | **(N = 56)** | **(N = 10)** | **(N = 46)** |  |
| **Onset-to-door time (min)** |  |  |  |  |
| n | 54 | 9 | 45 |  |
| Mean (SD) | 227.2 (335.7) | 317.7 (411.4) | 209.1 (320.9) | .47 |
| Median [Min, Max] | 76 [20, 1438] | 66 [25, 905] | 81 [20, 1438] | .69 |
| **Door-to-needle time (min)** |  |  |  |  |
| n | 51 | 8 | 43 |  |
| Mean (SD) | 73.6 (110.9) | 72.5 (65.7) | 73.8 (118.0) | .96 |
| Median [Min, Max] | 46 [3, 541] | 56 [12, 221] | 41 [3, 541] | .21 |
| **Onset-to-needle time (min)** |  |  |  |  |
| n | 53 | 9 | 44 |  |
| Mean (SD) | 267.4 (310.6) | 382.2 (426.5) | 243.9 (282.0) | .37 |
| Median [Min, Max] | 117 [25, 1390] | 115 [26, 999] | 121 [25, 1390] | .80 |
| **Length of stay (days)^b^** |  |  |  |  |
| n | 42 | 4 | 38 |  |
| Mean (SD) | 28.5 (21.0) | 27.0 (23.1) | 28.7 (21.1) | .90 |
| Median [Min, Max] | 24 [4, 89] | 22 [7, 58] | 24 [4, 89] | .80 |

**^a^** Wilcoxon rank-sum test and Welch’s two sample t-test were used for comparisons of medians and means, respectively.

**^b^** Length of stay was estimated among patients discharged alive after excluding in-hospital deaths, transfers to tertiary hospitals, and cases with missing data.

**Appendix F. Distribution of length of stay by group**

Regarding length of stay, the comparison group demonstrated both early and prolonged discharge patterns, whereas the intervention group showed a more gradual discharge pattern without extreme prolonged hospitalization (**Figure S6**).

Note: (A) includes the overall study population (n=163), whereas (B) excludes cases of in-hospital death and transfer to tertiary hospitals (n=131). (C) was based on the matched sample used in the GEE analysis in the main manuscript (n=91).

**Figure S6**. Kernel density plots of length of stay by analytic sample.
