# Supplementary material for: Digital Health–Enabled Prehospital Notification for Acute Stroke Using the Brain Saver Mobile App in Emergency Medical Transport in South Korea: Pilot Quasi-Experimental Study
Source: JMIR Med Inform. 2026 Oct 8;14:e92992. doi: 10.2196/92992 (PMC13691466; doi:10.2196/92992)
Supplement: Supplementary file 4 [file medinform_v14i1e92992_app4.docx]

This is a Multimedia Appendix to a full manuscript published in the *JMIR Medical Informatics.* For full copyright and citation information, see <http://dx.doi.org/10.2196/xxxxx>

**Brain Saver User Survey Results**

We surveyed users of both versions of the Brain Saver app: the emergency medical services (EMS) version, which has nine functions, and the clinician version, which has seven. Respondents were asked to rank up to three functions they found most satisfying, and some ranked fewer than three. We assigned 100, 70, and 30 points to first, second, and third place, respectively, and summed the points across respondents to obtain a weighted score for each function. Weighted percentages were calculated by dividing each function's weighted score by the total for all functions in that version and multiplying by 100.

Table S1 summarizes the EMS responses. Forty-two of 80 EMS personnel responded (response rate, 52.5%). One response was excluded because the respondent answered "yes" rather than ranking the functions, leaving 41 responses for analysis. Diagnostic result display was ranked highest, followed by distance-based hospital recommendation and automatic current-location entry.

Table S2 summarizes the clinician responses. Thirteen of 24 clinicians responded (response rate, 54.2%). Transport status tracking via the status bar was ranked highest, followed by viewing the patient diagnostic score and viewing the on-call clinician list within the same hospital.

**Table S1.** Survey results on Brain Saver functions among EMS personnel (N = 41)

| **Rank** | **Function** | **Number of rankings** | | | **Weighted score** | |
| --- | --- | --- | --- | --- | --- | --- |
|  |  | **1^st^**  **(n_1_)** | **2^nd^**  **(n_2_)** | **3^rd^**  **(n_3_)** | **Score (n_1_×w_1_**  **+n_2_×w_2_**  **+n_3_×w_3_)^a^** | **%^b^** |
| **1** | Diagnostic result display | 10 | 16 | 7 | 2,330 | 30.0% |
| **2** | Distance-based hospital recommendation | 6 | 8 | 9 | 1,430 | 18.4% |
| **3** | Automatic current-location entry | 12 | 1 | 2 | 1,330 | 17.1% |
| **4** | Real-time electrocardiogram sharing | 6 | 3 | 3 | 900 | 11.6% |
| **5** | Automatic transport-start alert | 5 | 2 | 6 | 820 | 10.6% |
| **6** | Transport status tracking via the status bar | 2 | 3 | 3 | 500 | 6.4% |
| **7** | Messaging | 0 | 3 | 2 | 270 | 3.5% |
| **8** | Final diagnosis confirmation after transport | 0 | 1 | 4 | 190 | 2.4% |
| **9** | Video call | 0 | 0 | 0 | 0 | 0.0% |
| **Total** | | 41 | 37 | 36 | 7,770 | 100.0% |

ᵃ Weights of 100 (w₁), 70 (w₂), and 30 (w₃) were assigned to first-, second-, and third-ranked responses, respectively.

ᵇ Weighted score of each function divided by the sum of weighted scores for all functions, multiplied by 100.

**Table S2.** Survey results on Brain Saver functions among clinicians (N = 13)

| **Rank** | **Function** | **Number of rankings** | | | | **Weighted score** | |
| --- | --- | --- | --- | --- | --- | --- | --- |
|  |  | **1^st^**  **(n_1_)** | **2^nd^**  **(n_2_)** | **3^rd^**  **(n_3_)** | **Score (n_1_×w_1_**  **+n_2_×w_2_**  **+n_3_×w_3_)^a^** | | **%^b^** |
| **1** | Transport status tracking via the status bar | 6 | 4 | 1 | 910 | | 39.6% |
| **2** | Viewing the patient diagnostic score | 5 | 3 | 0 | 710 | | 30.9% |
| **3** | Viewing the on-call clinician list within the same hospital | 2 | 0 | 1 | 230 | | 10.0% |
| **4** | Real-time electrocardiogram sharing | 0 | 1 | 3 | 160 | | 7.0% |
| **5** | Messaging | 0 | 1 | 2 | 130 | | 5.7% |
| **6** | Arrhythmia alarm | 0 | 1 | 1 | 100 | | 4.3% |
| **7** | Video call | 0 | 0 | 2 | 60 | | 2.6% |
| **Total** | | 13 | 10 | 10 | 2,300 | | 100.0% |

ᵃ Weights of 100 (w₁), 70 (w₂), and 30 (w₃) were assigned to first-, second-, and third-ranked responses, respectively.

ᵇ Weighted score of each function divided by the sum of weighted scores for all functions, multiplied by 100.
